# Evaluation of patients treated with direct-acting anti-viral therapy for chronic hepatitis C and their risk of hepatocellular carcinoma in Hong Kong

**DOI:** 10.1186/s12876-023-03099-2

**Published:** 2024-01-25

**Authors:** Victor Yung Sin Chow, Wing I Cheung

**Affiliations:** Our Lady of Maryknoll Hospital, Hong Kong, China

**Keywords:** Hepatitis C, Direct-acting anti-viral therapy, Hepatocellular carcinoma

## Abstract

**Background & aim:**

To evaluate the risk of early hepatocellular carcinoma (HCC) in chronic hepatitis C patients treated with direct-acting antivirals (DAAs) in Hong Kong, as it has not been studied before in this locality.

**Methods:**

Three hundred thirty-three consecutive chronic hepatitis C patients treated with DAAs from two hospitals over the past 6 years were identified. Kaplan-Meier method was used to calculate cumulative HCC incidence. Cox regression was used to identify factors associated with HCC development.

**Results:**

During a median follow-up of 23.4 months after DAA started, 15 (5.4%, 95% CI 3.3–8.7%) out of 279 total included patients developed HCC. The overall sustained virological response (SVR) rate was 98.9%. The 1-year cumulative incidence for de-novo HCC and HCC recurrence were 0.8 and 30.9%, respectively (log-rank test *p* < 0.001). The 1-year cumulative HCC incidence for patients without and with cirrhosis were 0.7 and 5.1%, respectively (log-rank test *p* = 0.036). Univariate analysis showed that significant factors associated with HCC after DAA were: history of treated HCC, cirrhosis, evidence of portal hypertension, higher AFP at the start or end of DAA therapy, higher bilirubin, lower platelets, lower albumin, and older age. From receiver operating characteristic curve analysis, the optimal cut-off level of AFP for predicting HCC was 10.5 ng/mL at the start and 5.6 ng/mL at the end of DAA therapy.

**Conclusions:**

The risk of early HCC recurrence remains high despite achieving SVR following DAA therapy, whereas the risk of early de-novo HCC occurence is low. AFP levels, both at the start and end of DAA therapy, can be useful in stratifying risks of HCC development.

**Supplementary Information:**

The online version contains supplementary material available at 10.1186/s12876-023-03099-2.

## Introduction

According to data from the World Health Organization, an estimated 71 million people are chronically infected with the hepatitis C virus (HCV) [1]. This is equivalent to 1% of the global population. In Hong Kong, the seroprevalence rate of HCV infection is 0.3% [2, 3]. Multiple studies have shown that 5–20% of patients with chronic HCV would progress to advanced fibrosis and cirrhosis over a period of 5–20 years [4, 5]. Once HCV-induced cirrhosis has been established, the annual risk of developing hepatocellular carcinoma (HCC) is 3–7%, making chronic HCV infection the leading risk factor for HCC [6, 7]**.** In fact, of all patients infected with chronic HCV, without treatment, 14.4% would develop HCC according to forecasting models [8]. HCC, being the most common histologic type among primary liver neoplasia, is the seventh most common cancer and the fourth leading cause of cancer-related death globally [9, 10]. Curative treatments for HCC include surgical resection, thermal ablation, and liver transplantation [11]. However, despite these curative treatments, the annual HCC recurrence rate can be up to 15–20% [12, 13], and the recurrence risk can increase up to 75% after 5 years of intervention [14, 15]. Intra-hepatic HCC recurrence can be classified into early (within 2 years) and late (after 2 years) recurrence [16].

Direct-acting anti-viral (DAA) therapy has revolutionized the treatment landscape of HCV infection. These interferon(IFN)-free regimens can achieve sustained virological response(SVR) rates as high as 95–98% [17]. It has been shown that DAA therapy can lower portal hypertension, improve liver dysfunction and induce fibrosis regression after achieving SVR [18–21]. Therefore, it was reasonable to expect that DAA-induced SVR would reduce the risk of HCC by preventing cirrhosis, or by eliminating the carcinogenic effect of HCV. Interestingly, since 2016, several studies have reported unexpectedly high rates of de-novo HCC occurrence and HCC recurrence after DAA therapy [22–24]. In a multi-center retrospective Spanish study [24], Reig et al. found that 27.6% of patients developed HCC recurrence over a median follow-up of 5.7 months after DAA therapy for chronic hepatitis C. Although this study was then heavily criticized for its methodologies, such as the short follow-up period and the small sample size of only 58 patients, the alarmingly high rate of HCC recurrence certainly did cause concern over the use of DAA therapy. This has sparked debate over the past 6 years on the topic – would DAA therapy for the treatment of chronic HCV infection be associated with an increased risk of de-novo HCC occurrence and/or HCC recurrence?

The main purpose of this study was to investigate the risk of early HCC development in a cohort of chronic hepatitis C patients treated with DAA therapy in Hong Kong. Risk factors for HCC in this group of patients would be determined. Other poor clinical outcomes including other liver-related complications and death were also studied.

## Methods

### Study design

Consecutive patients from the Princess Margaret Hospital and Our Lady of Maryknoll Hospital with chronic hepatitis C who were treated with a course of DAA therapy, with a treatment start date between 1st August 2015 and 31st July 2021, were retrospectively recruited into the study. For patients with a history of HCC before initiation of DAA therapy, additional inclusion criteria were: i) previous HCC diagnosed by pathology or non-invasively by radiological imaging according to the American Association for the Study of Liver Diseases and European Association for the Study of the Liver guidelines [11, 25]; ii) completion of HCC treatment either by surgical resection, ablation and/or trans-arterial(chemo-)embolization; iii) documented complete radiological response to HCC treatment prior initiation of DAA therapy. The exclusion criteria for all patients were: i) less than 18-year-old; ii) history of liver transplantation; iii) presence of non-characterized liver nodule(s) radiologically before initiation of DAA therapy; iv) never received radiological imaging of the abdomen; v) patients who did not complete the full course of DAA therapy; vi) lost to follow-up, unknown treatment outcome or had no evaluable SVR data; vii) received IFN as part of the anti-viral treatment regimen; viii) presence of active HCC before initiation of DAA therapy.

### Outcomes measure

The main outcomes were i) de-novo HCC occurrence after the initiation of DAA therapy in chronic hepatitis C patients; ii) HCC recurrence after the initiation of DAA therapy in chronic hepatitis C patients with a history of HCC previously who have achieved complete radiologic response following HCC curative treatment.

Secondary outcomes were the development of other liver-related complication(s) in previously compensated cirrhosis and/or all-cause mortality after the initiation of DAA therapy.

### Definitions

DAA regimens include sofosbuvir, sofosbuvir plus ledipasvir, sofosbuvir plus velpatasvir, sofosbuvir plus velpatasvir plus voxilaprevir, glecaprevir plus pibrentasvir, asunaprevir plus daclatasvir, elbasvir plus grazoprevir, and ombitasvir plus paritaprevir plus ritonavir plus dasabuvir; with or without ribavirin as indicated.

De-novo HCC was defined as the occurrence of new HCC in patients without a history of HCC previously.

HCC recurrence was defined as the new development of HCC in patients with a history of HCC previously who have already achieved complete radiologic response following previous curative HCC treatment.

The diagnosis of cirrhosis would be established if at least one of the following was present: i) liver histology showing stage 4 fibrosis (METAVIR score); ii) liver stiffness measurement (LSM) greater than 12.5 kPa at transient elastography (FibroScan, Echosens, Paris, France) [26–32]; iii) signs of cirrhosis on imaging of the abdomen [33]; iv) evidence of portal hypertension at Oesophago-gastro-duodenoscopy (OGD). The Child-Pugh score was used to determine the functional class of cirrhosis.

Evidence of portal hypertension includes the presence of oesophageal varices, gastric varices, splenomegaly, portal hypertensive gastropathy, or splenic varices.

Sustained virological response (SVR) was defined as undetectable HCV-RNA from the serum at least 12 weeks after the end of DAA treatment. HCV-RNA quantification was assessed by real-time PCR, with a limit of detection of 15 IU/mL.

Complete radiologic response (CRR) of HCC was defined as the absence of residual tumor or complete necrosis of HCC, assessed by Computed Tomography (CT) scan or Magnetic Resonance Imaging (MRI) [11].

Among the patients with chronic hepatitis C, hepatitis B (HBV) co-infection was defined as either a positive HBV surface antigen test or detectable HBV DNA from the serum. Human immunodeficiency virus (HIV) co-infection was defined as either a positive HIV antibody test or detectable HIV viral load from the serum.

Other liver-related complication(s) was defined as the occurrence of ascites, hepatic encephalopathy, variceal haemorrhage, spontaneous bacterial peritonitis, hepato-renal syndrome, or portal vein thrombosis (not including HCC in the current study).

### Statistical analysis

Baseline variables reported in Table 1 were assessed as potential risk factors for the outcomes. Categorial variables were presented as frequencies and percentages while continuous variables were presented as means +/− standard deviations or median and range. The Chi-square test or the Fisher’s exact test, where appropriate, was used to compare categorical variables. The Student’s t-test or the Mann-Whitney test, where appropriate, was used to compare continuous variables. The curves showing the cumulative incidence of HCC and other liver-related complications during the follow-up period were drawn using the Kaplan-Meier (KM) method. The follow-up period for the main outcomes was defined as the time between the start date of DAA therapy and either i) the date of diagnosis of HCC occurrence/recurrence radiologically; ii) the latest imaging date for those who did not newly develop HCC after DAA therapy; iii) death; or iv) the end date of the current study - 15th January 2022; whichever was earlier. Differences among cohorts of patients were assessed using the log-rank test. Univariate Cox regression analysis was used to evaluate baseline variables as risk factors for HCC development and to generate their hazard ratios (HR) and 95% confidence intervals (CI). Receiver operating characteristic (ROC) curve analysis was performed to determine the optimal cut-off level of AFP for predicting HCC development, both at the start and end of DAA therapy. The optimal cut-off level was selected from the ROC curve using the point closest to the top left method. The area under the ROC curve was calculated with 95% CI for the sensitivity and specificity of the AFP cut-off level. *P* < .05 was considered statistically significant in all analyses. All *p*-values were two-tailed and all confidence intervals were 95%. The software SPSS Statistics version 26.0 (IBM SPSS Inc. Chicago, IL, USA) was used to conduct statistical analyses and plot results. For missing data, no imputation was performed.

**Table 1:** Baseline characteristics of the entire study population

	Total		No history of HCC before DAA		History of treated HCC with CRR before DAA		*p*-value
	(*n* = 279)		(*n* = 261)		(*n* = 18)		
**Male**	183	(65.6)	168	(64.4)	15	(83.3)	0.101
**Age, year**	59.0	± 12.8	58.3	± 12.8	68.9	± 9.4	**< 0.001**
**BMI, kg/m**^**2**^	23.7	± 4.0	23.7	± 4.0	23.6	± 3.8	0.959
**HCV genotype**							0.126
**1a**	13	(4.7)	12	(4.6)	1	(5.6)	
**1b**	136	(49.1)	127	(49.0)	9	(50)	
**2**	7	(2.5)	5	(1.9)	2	(11.1)	
**3**	22	(7.9)	20	(7.7)	2	(11.1)	
**6**	99	(35.7)	95	(36.7)	4	(22.2)	
**HCV viral load before DAA therapy, × 10**^**6**^ **IU/ml**	3.4	± 9.1	3.6	± 9.4	1.6	± 1.9	0.129
**DAA therapy duration, weeks**	12 [8, 12]		12 [8, 12]		12 [12, 12]		0.071
**Haemoglobin, g/dL**	13.8	± 2.0	13.8	± 2.0	13.2	± 1.9	0.202
**Platelets, × 10**^**9**^**/L**	172.0	± 71.3	175.7	± 71.1	119.8	± 52.1	**< 0.001**
**Creatinine, umol/L**	85.0	± 84.6	85.1	± 87.2	83.7	± 27.1	0.333
**ALT at the start of DAA therapy, IU/L**	85.9	± 109.9	85.7	± 111.8	87.6	± 79.0	0.919
**ALT at the end of DAA therapy, IU/L**	24.1	± 19.2	24.2	± 19.7	22.4	± 7.9	0.408
**Total bilirubin, umol/L**	13.4	± 7.1	12.9	± 6.4	20.5	± 11.8	**0.005**
**Albumin, g/L**	37.9	± 5.2	38.2	± 5.0	33.6	± 5.6	**< 0.001**
**INR**	1.06	± 0.08	1.06	± 0.08	1.14	± 0.12	**< 0.001**
**Prothrombin time, second**	11.7	± 1.1	11.6	± 1.1	12.8	± 1.5	**< 0.001**
**AFP at the start of DAA therapy, ng/ml**	11.1	± 25.6	10.0	± 24.7	26.4	± 33.2	**< 0.001**
**AFP at the end of DAA therapy, ng/ml**	4.5	± 5.3	4.1	± 5.0	9.2	± 6.9	**< 0.001**
**HBV co-infection**	16	(5.7)	16	(6.1)	0		0.609
**HIV co-infection**	20	(7.2)	20	(7.7)	0		0.627
**Diabetes mellitus**	49	(17.6)	48	(18.4)	1	(5.6)	0.214
**Cirrhosis**	131	(47.0)	115	(44.1)	16	(88.9)	**< 0.001**
**Liver stiffness, kPa**	10.3 [7.1, 14.975]		10.1 [7.05, 14.5]		17.5 [12.1, 24.5]		**0.004**
**Fatty liver**	103	(36.9)	100	(38.3)	3	(16.7)	0.066
**SVR 12**	276	(98.9)	258	(98.9)	18	(100)	1
**Evidence of portal hypertension**	65	(23.3)	54	(20.7)	11	(61.1)	**< 0.001**
**History of anti-viral treatment before DAA therapy**	74	(26.5)	69	(26.4)	5	(27.8)	1
**HCV infection suspected to be via previous intra-venous drug abuse**	119	(42.7)	113	(43.3)	6	(33.3)	0.409
**HCV infection suspected to be via previous blood transfusion**	60	(21.5)	59	(22.6)	1	(5.6)	0.135
**Time between latest imaging (before DAA) till DAA start date, months**	15.5	± 22.6	16.1	± 23.2	6.7	± 7.9	0.059
**AFP at diagnosis of previous HCC before DAA therapy, ng/ml**	–		–		90.2	± 104.8	–
**Previous HCC nodule before DAA therapy:**							
**Single**	–		–		16	(88.9)	–
**Multiple**	–		–		1	(5.6)	–
**Maximum HCC nodule size (before DAA therapy), cm**	–		–		2.7	± 1.6	–
**Previous HCC treatment before DAA therapy:**							
**Resection**	–		–		4	(22.2)	–
**Ablation ± TACE**	–		–		14	(77.8)	–
**Time between last HCC treatment till latest imaging before DAA, months**	–		–		14.9	± 24.5	–
**Time between last HCC treatment till DAA therapy start, months**	–		–		21.5	± 30.7	–

Data was expressed as count (%), mean ± SD, or median [IQR]

*AFP* alfa-fetoprotein, *ALT* alanine transaminase, *BMI* Body-mass index, *DAA* direct-acting anti-viral, *HBV* hepatitis B virus, *HCC* hepatocellular carcinoma, *HCV* hepatitis C virus, *HIV* human immunodeficiency virus, *INR* international normalized ratio, *SVR* sustained virological response

## Results

### Baseline characteristics

**Fig. 1:**
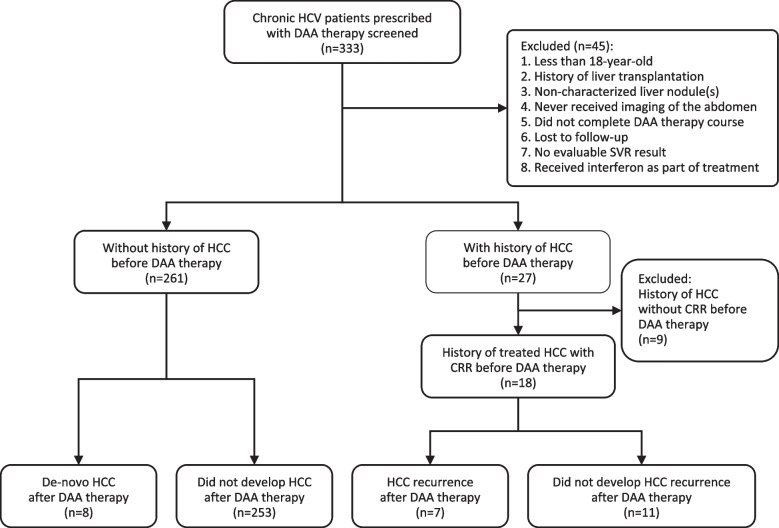
Flow chart of the patient selection process. CRR, complete radiologic response. DAA, direct-acting anti-viral therapy. HCC, hepatocellular carcinoma. SVR, sustained virological response

333 consecutive patients (284 patients from the Princess Margaret Hospital, and 49 patients from the Our Lady of Maryknoll Hospital) from the two hospitals with chronic hepatitis C were prescribed with a course of DAA therapy during the study period. A flow chart of the patient selection process is shown in Fig. 1**.**

None of the patients was less than 18-year-old and none has received a liver transplant previously. 3 patients were excluded for having an uncharacterized liver nodule before the start of DAA therapy. 14 patients were excluded as they have never received an abdominal imaging before or after DAA therapy. 5 patients were excluded because they did not complete the whole DAA course. 21 patients were excluded as they were either lost to follow-up or had no evaluable SVR data. 2 patients were excluded since their anti-viral treatment regimen were a combination of IFN and sofosbuvir. 9 patients were excluded as they had active HCC or ongoing HCC treatment at the start of DAA therapy.

The remaining 279 patients (245 patients from the Princess Margaret Hospital, and 34 patients from the Our Lady of Maryknoll Hospital) were included in the subsequent analyses. Their demographic and baseline characteristics are depicted in Table 1**.** Of all the included patients, the mean age was 59 ± 12.8 years, 65.6% were male and 73.5% were HCV treatment-naïve. A predominant 49.1% of them were infected with genotype 1b, 35.7% were genotype 6, and 7.9% were genotype 3. The overall SVR rate was 98.9%. 47% had cirrhosis, of which 89.3% were Child-Pugh class A and the rest were Child-Pugh class B, at the start of DAA therapy. 65 out of the 131 (49.6%) patients with cirrhosis had evidence of portal hypertension. Only 5.7% had hepatitis B co-infection and 7.2% had HIV co-infection. 1 patient had triple viral infections but despite that, the patient never developed HCC before or after DAA therapy. 17.6% had diabetes mellitus and their mean HbA1c at the start of DAA therapy was 7.1%**.** 36.9% had fatty liver diagnosed, either radiologically or by transient elastography. The source of infection of chronic HCV was thought to be via intra-venous drug abuse in 42.7% and via blood transfusion in 21.5% of all the included patients.

18 patients (6.5%) had a history of HCC with previous curative treatment, and confirmed complete radiologic response before the start of DAA therapy.

### Main outcomes

During an overall mean follow-up period of 28.2 months after the initiation of DAA therapy (median 23.4 months, inter-quartile range 14.2–37.1 months) in the whole cohort, 15 (5.4%) out of 279 patients developed HCC (Table 2).

**Table 2:** Outcomes after DAA therapy

	Count	Rate (95% CI)
**Development of HCC**	15/279	5.4% (3.3–8.7%)
**De-novo HCC occurrence**	8/261	3.1% (1.6–5.9%)
**HCC recurrence**	7/18	38.9% (20.3–61.4%)
**Newly developed other liver-related complications**	24/279	8.6% (5.8–12.5%)
**Ascites**	13	
**Hepatic encephalopathy**	7	
**Spontaneous bacterial peritonitis**	2	
**Hepato-renal syndrome**	1	
**Variceal haemorrhage**	4	
**Portal vein thrombosis**	6	
**All-cause mortality**	12/279	4.3% (2.5–7.4%)

Univariate cox regression analysis showed that higher bilirubin (HR 1.09, 95% CI 1.04–1.14, *p* < 0.001), higher AFP at the start of DAA therapy (HR 1.02, 95% CI 1.01–1.03, *p* < 0.001), higher AFP at the end of DAA therapy (HR 1.09, 95% CI 1.05–1.14, *p* < 0.001) and history of previous HCC despite curative treatment with confirmed complete radiologic response (HR 28.88, 95% CI 9.32–89.49, *p* < 0.001), were all strongly significant factors associated with the development of HCC after DAA therapy. Other significant factors include more advanced age, lower platelets, lower albumin, presence of cirrhosis, and evidence of portal hypertension. Known risk factors for HCC such as being male, genotype 1b, HBV co-infection, HIV co-infection, or diabetes mellitus did not reach statistical significance. The details are shown in Table 3.

**Table 3:** Factors associated with HCC development after DAA therapy: Univariate Cox regression analysis

	Did not develop HCC after DAA therapy		Developed HCC after DAA therapy		Univariate		
	(*n* = 264)		(*n* = 15)		HR_unadj_(95% CI)		*p*-value
**History of treated HCC with CRR before DAA therapy start**	11	(4.2)	7	(46.7)	28.88	(9.32–89.49)	**< 0.001**
**Male**	171	(64.8)	12	(80)	2.47	(0.69–8.78)	0.162
**Age, year**	58.5	± 12.9	67.1	± 9.4	1.07	(1.02–1.12)	**0.007**
**BMI, kg/m**^**2**^	23.6	± 4.0	24.6	± 3.9	1.06	(0.92–1.22)	0.400
**HCV genotype**							
**1b**	126	(47.7)	10	(66.7)	1.78	(0.61–5.22)	0.295
**Non-1b**	136	(51.5)	5	(33.3)	1		–
**HCV viral load before DAA therapy, ×10**^**6**^ **IU/ml**	3.5	± 9.4	2.4	± 2.5	0.97	(0.82–1.13)	0.663
**DAA therapy duration, weeks**	12 [8, 12]		12 [12, 12]		1.07	(0.96–1.18)	0.210
**Haemoglobin, g/dL**	13.8	± 2.0	14.1	± 2.1	1.12	(0.86–1.47)	0.410
**Platelets, ×10**^**9**^**/L**	174.6	± 71.4	128.4	± 56.8	0.99	(0.98–0.999)	**0.035**
**Creatinine, umol/L**	85.0	± 86.7	84.9	± 30.8	1.00	(0.99–1.01)	0.901
**ALT at the start of DAA therapy, IU/L**	85.3	± 111.3	96.2	± 83.7	1.001	(0.997–1.004)	0.703
**ALT at the end of DAA therapy, IU/L**	23.6	± 17.9	31.3	± 34.7	1.01	(0.99–1.03)	0.209
**Total bilirubin, umol/L**	13.0	± 6.5	20.5	± 12.1	1.09	(1.04–1.14)	**< 0.001**
**Albumin, g/L**	38.1	± 5.0	35.1	± 7.0	0.89	(0.81–0.97)	**0.007**
**Prothrombin time, second**	11.7	± 1.1	12.0	± 1.3	1.32	(0.86–2.02)	0.202
**AFP at the start of DAA therapy, ng/ml**	9.0	± 18.6	47.2	± 70.1	1.02	(1.01–1.03)	**< 0.001**
**AFP at the end of DAA therapy, ng/ml**	4.1	± 5.1	10.0	± 6.6	1.09	(1.05–1.14)	**< 0.001**
**HBV co-infection**	15	(5.7)	1	(6.7)	0.99	(0.13–7.57)	0.990
**HIV co-infection**	20	(7.6)	0		–		–
**Diabetes mellitus**	47	(17.8)	2	(13.3)	0.64	(0.14–2.83)	0.554
**Cirrhosis**	119	(45.1)	12	(80)	3.57	(1.00–12.77)	**0.0499**
**Liver stiffness, kPa**	10.1 [7, 14.5]		22.3 [14.4, 26.6]		1.03	(1.00–1.06)	0.079
**Fatty liver**	101	(38.3)	2	(13.3)	0.26	(0.06–1.17)	0.079
**SVR 12**	261	(98.9)	15	(100)	–		–
**Evidence of portal hypertension**	56	(21.2)	9	(60)	4.27	(1.51–12.07)	**0.006**
**History of anti-viral treatment before DAA therapy**	68	(25.8)	6	(40)	1.18	(0.40–3.43)	0.766
**HCV infection suspected to be via previous intra-venous drug abuse**	113	(42.8)	6	(40)	1.11	(0.39–3.13)	0.848
**HCV infection suspected to be via previous blood transfusion**	57	(21.6)	3	(20)	0.69	(0.19–2.46)	0.568

Data was expressed as count (%), mean ± SD, or median [IQR]

*AFP* alfa-fetoprotein, *ALT* alanine transaminase, *BMI* Body-mass index, *DAA* direct-acting anti-viral, *HBV* hepatitis B virus, *HCC* hepatocellular carcinoma, *HCV* hepatitis C virus, *HIV* human immunodeficiency virus, *HR* hazard ratio, *INR* international normalized ratio, *SVR* sustained virological response

Bold *p*-values are < 0.05, i.e. statistically significant

**Fig. 2:**
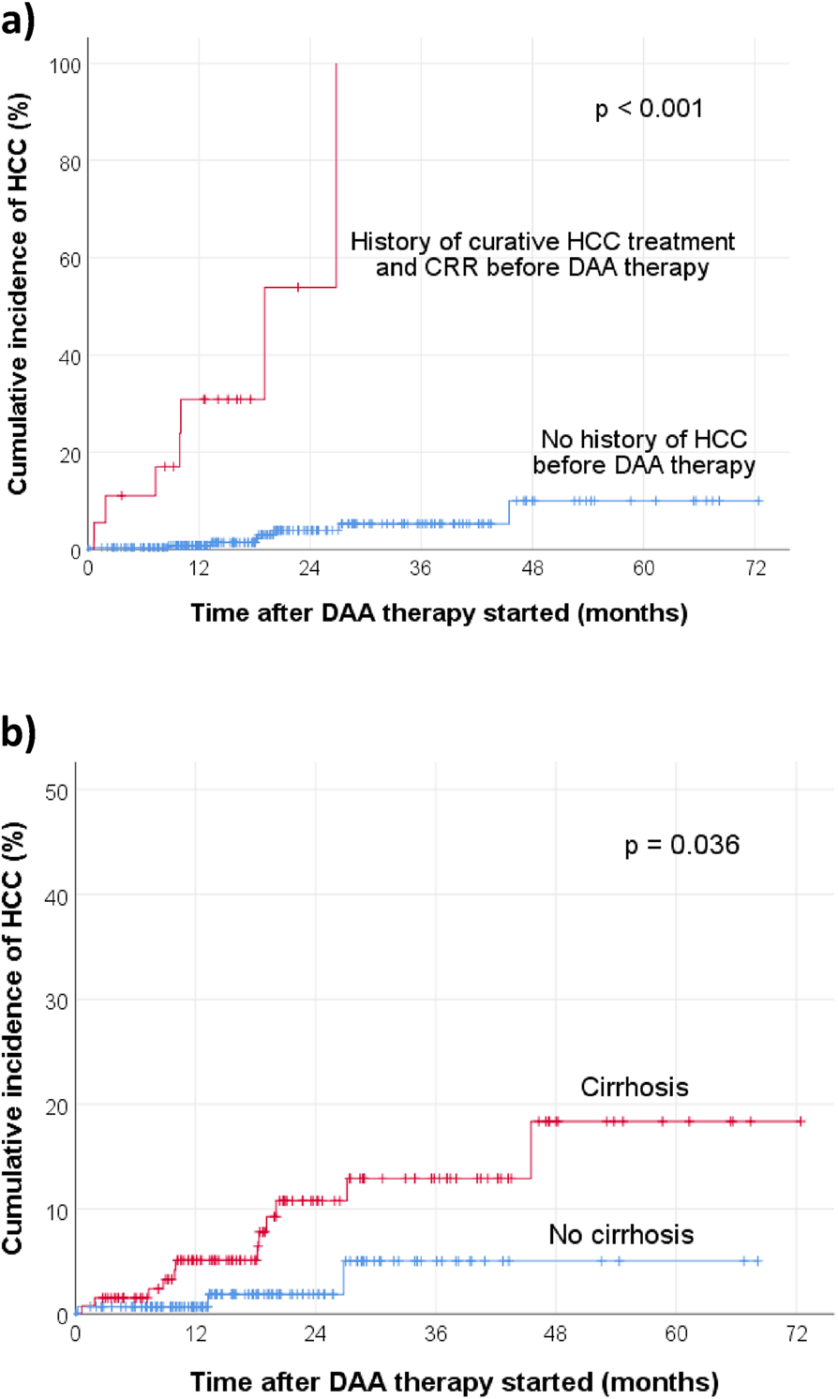
Cumulative incidence of HCC after the initiation of DAA therapy, **a** stratified by the presence or absence of previous HCC before DAA therapy; **b** stratified by the presence or absence of cirrhosis. CRR, complete radiologic response

After the initiation of DAA therapy, the 1-year cumulative incidence of HCC for de-novo development and recurrence was 0.8% (95% CI 0–2%) and 30.9% (95% CI 8–53.7%), respectively (log-rank test p < 0.001). The KM curves are shown in Fig. 2a. After the initiation of DAA therapy, the 1-year cumulative incidence of HCC development for patients without and with cirrhosis was 0.7% (95% CI 0–2%) and 5.1% (95% CI 1.1–9.1%), respectively (log-rank test *p* = 0.036). The KM curves are shown in Fig. 2b.

**Fig. 3:**
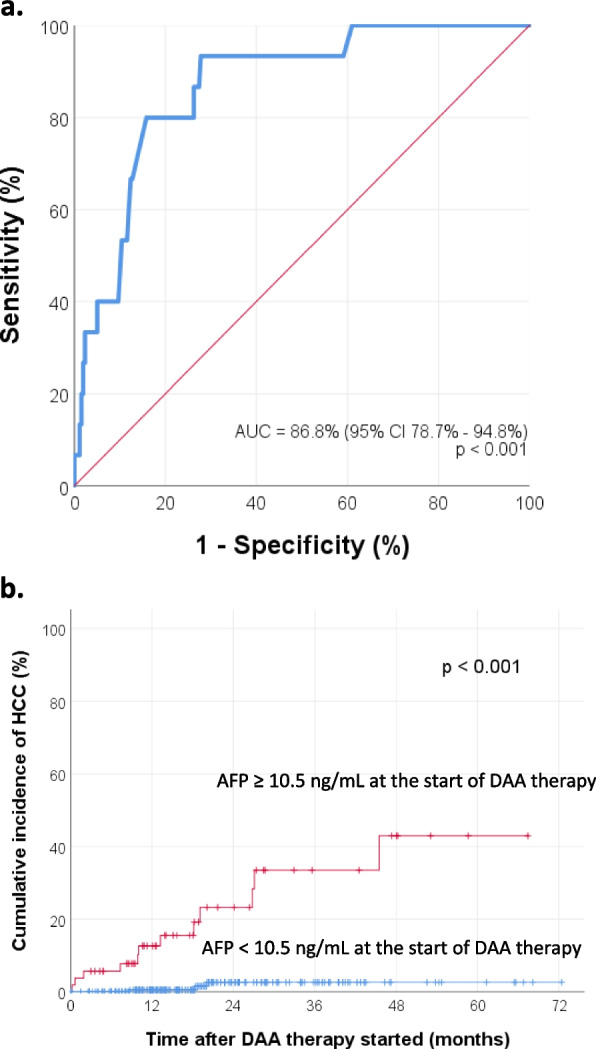
**a** ROC curve analysis shows that the optimal cut-off level of AFP at the start of DAA therapy for predicting HCC development was 10.5 ng/mL (sensitivity 80%, specificity 84.2%, Area under the ROC curve = 0.868). **b** Cumulative incidence of HCC, stratified by the AFP level at the start of DAA therapy

**Fig. 4:**
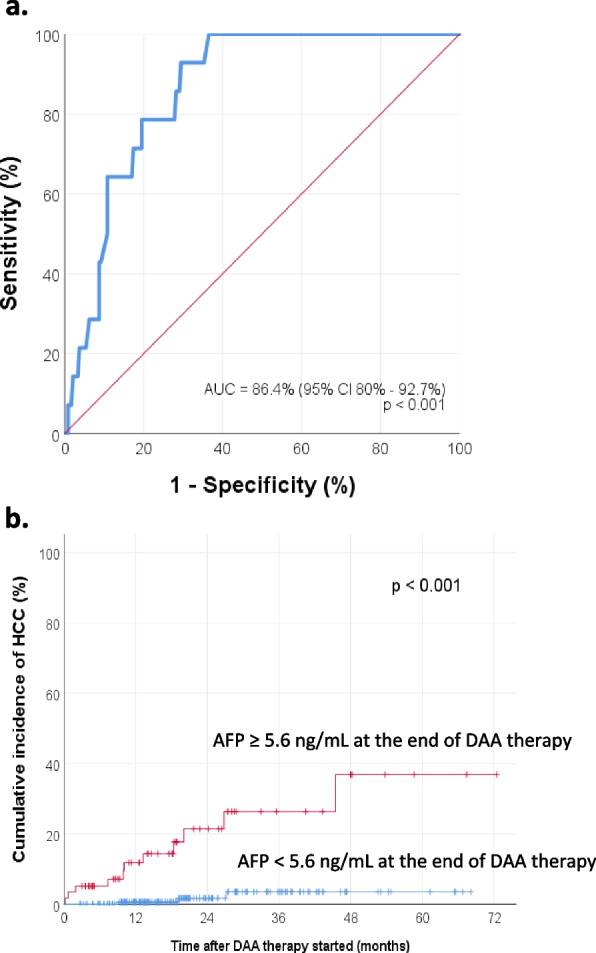
**a** ROC curve analysis shows that the optimal cut-off level of AFP at the end of DAA therapy for predicting HCC development was 5.6 ng/mL (sensitivity 78.6%, specificity 80.5%, Area under the ROC curve = 0.864). **b** Cumulative incidence of HCC, stratified by the AFP level at the end of DAA therapy

Receiver operating characteristic (ROC) curve analysis was then performed to determine the optimal cut-off levels of AFP for predicting HCC development. The optimal cut-off level of AFP at the start of DAA therapy was 10.5 ng/mL (sensitivity 80%, specificity 84.2%, AUROC 0.868), shown in Fig. 3a. The cumulative incidence of HCC development for the group with AFP level < 10.5 ng/mL at the start of DAA therapy was significantly lower than that for the group with AFP level ≥ 10.5 ng/mL (log-rank test *p* < 0.001). The KM curves are shown in Fig. 3b. The 1-year cumulative incidence of HCC were 0.5% (95% CI 0–1.6%) and 12.6% (95% CI 3.1–22.1%) for the groups with AFP level < 10.5 ng/mL and ≥ 10.5 ng/mL at the start of DAA therapy, respectively. On the other hand, the optimal cut-off level of AFP at the end of DAA therapy was 5.6 ng/mL (sensitivity 78.6%, specificity 80.5%, AUROC 0.864), shown in Fig. 4a. The cumulative incidence of HCC development for the group with AFP level < 5.6 ng/mL at the end of DAA therapy was significantly lower than that for the group with AFP level ≥ 5.6 ng/mL (log-rank test *p* < 0.001). The KM curves are shown in Fig. 4b. The 1-year cumulative incidence of HCC were 0.6% (95% CI 0–1.7%) and 11.8% (95% CI 2.8–20.8%) for the groups with AFP level < 5.6 ng/mL and ≥ 5.6 ng/mL at the end of DAA therapy, respectively.

### Virological response

Although multiple studies have previously shown that lack of SVR was a strong predictor of HCC development [34, 35], in the current study all the patients who developed de-novo HCC occurrence or HCC recurrence had achieved SVR. The SVR rate for those who did not develop HCC was 98.9%. Of the 3 patients who did not achieve SVR, 2 of them were infected with genotype 3, which is a known predictor of lower treatment response, compared to other genotypes [36, 37]. None of these 3 patients developed HCC, other liver-related complications or died by the end of the follow-up period. None of the SVR patients had evidence of late relapse or re-infection after week 12 post-DAA therapy.

### Sub-group analysis for patients without a history of HCC before DAA therapy

8 out of 261 patients (3.1%) developed de-novo HCC after the start of DAA therapy. Details of their individual and HCC characteristics are shown in the Supplementary Table 1. None of the de-novo HCC had major vessel or extra-hepatic involvement. All of them were within the Milan criteria. The mean time from the last imaging confirming the absence of active HCC before DAA therapy till the DAA start date was 2.6 months. The mean time from the start of DAA therapy till development of de-novo HCC was 18.9 months.

As shown in Table 4, analysis of the clinical characteristics showed that patients who developed de-novo HCC were significantly older (*p* = 0.044), had lower creatinine (*p* = 0.039), higher AFP at the start of DAA therapy (*p* = 0.001), higher AFP at the end of DAA therapy (*p* = 0.002), and higher pre-DAA liver stiffness measurement as assessed by transient elastography (*p* = 0.009), compared to those who have never developed HCC. The two groups did not have significant difference in terms of gender, body mass index, genotypes, HCV RNA viral load, ALT level, DAA therapy duration, presence of diabetes mellitus, fatty liver, SVR rate, or previous anti-viral treatment history.

**Table 4:** Sub-group analysis for patients without a history of HCC before DAA therapy

	Did not develop HCC after DAA therapy		De-novo HCC after DAA therapy		*p*-value
	(*n* = 253)		(*n* = 8)		
**Male**	163	(64.4)	5	(62.5)	1
**Age, year**	58.0	± 12.8	67.3	± 7.6	**0.044**
**BMI, kg/m**^**2**^	23.7	± 4.0	23.1	± 2.7	0.806
**HCV genotype**					0.785
**1a**	12	(4.7)	0		
**1b**	121	(47.8)	6	(75)	
**2**	5	(2.0)	0		
**3**	20	(7.9)	0		
**6**	93	(36.8)	2	(25)	
**HCV viral load before DAA therapy, ×10**^**6**^ **IU/ml**	3.6	± 9.5	3.7	± 2.6	0.154
**DAA therapy duration, weeks**	12 [8, 12]		12 [12, 12]		0.154
**Haemoglobin, g/dL**	13.8	± 2.0	14.6	± 2.3	0.092
**Platelets, ×10**^**9**^**/L**	177.0	± 71.4	134.3	± 51.2	0.337
**Creatinine, umol/L**	85.3	± 88.4	78.3	± 33.1	**0.039**
**ALT at the start of DAA therapy, IU/L**	84.8	± 112.3	115.4	± 97.7	0.415
**ALT at the end of DAA therapy, IU/L**	23.7	± 18.3	40.3	± 46.0	0.155
**Total bilirubin, umol/L**	12.8	± 6.5	14.9	± 5.7	0.159
**Albumin, g/L**	38.2	± 5.0	39.3	± 3.5	0.216
**INR**	1.06	± 0.08	1.01	± 0.04	0.492
**Prothrombin time, second**	11.6	± 1.1	11.1	± 0.6	0.059
**AFP at the start of DAA therapy, ng/ml**	8.3	± 17.0	64.3	± 92.8	**0.001**
**AFP at the end of DAA therapy, ng/ml**	4.0	± 5.1	6.8	± 1.8	**0.002**
**HBV co-infection**	15	(5.9)	1	(12.5)	0.401
**HIV co-infection**	20	(7.9)	0		1
**Diabetes mellitus**	46	(18.2)	2	(25)	0.642
**Cirrhosis**	109	(43.1)	6	(75)	0.144
**Liver stiffness, kPa**	9.9 [7, 14.2]		22.3 [14.4, 26.6]		**0.009**
**Fatty liver**	98	(38.7)	2	(25)	0.714
**SVR 12**	250	(98.8)	8	(100)	1
**Evidence of portal hypertension**	50	(19.8)	4	(50)	0.06
**History of anti-viral treatment before DAA therapy**	65	(25.7)	4	(50)	0.214
**HCV infection suspected to be via previous intra-venous drug abuse**	109	(43.1)	4	(50)	0.730
**HCV infection suspected to be via previous blood transfusion**	56	(22.1)	3	(37.5)	0.386

Data was expressed as count (%), mean ± SD or median [IQR]

*AFP* alfa-fetoprotein, *ALT* alanine transaminase, *BMI* Body-mass index, *DAA* direct-acting anti-viral, *HBV* hepatitis B virus, *HCC* hepatocellular carcinoma, *HCV* hepatitis C virus, *HIV* human immunodeficiency virus, *INR* international normalized ratio, *SVR* sustained virological response

Bold *p*-values are < 0.05, i.e. statistically significant

### Sub-group analysis for patients with a history of curative HCC treatment and confirmed complete radiologic response before DAA therapy

18 patients were in this sub-group. Regarding their previous HCC prior DAA therapy, 7 were at BCLC stage 0, 9 were at BCLC stage A and 1 was at BCLC stage B. 1 of them had missing data of the characteristics of the previous HCC, for which the patient received radiofrequency ablation. This patient did not develop HCC recurrence after DAA therapy by the end of the study period. Details of their previous HCC characteristics and the treatments they received before initiation of DAA therapy are shown in the Supplementary Table 1.

7 out of the 18 patients in this sub-group (38.9%) developed HCC after the start of DAA therapy, i.e. HCC recurrence. The time from the date of last treatment for previous HCC till DAA therapy start date was not significantly different between those who developed HCC recurrence versus those who did not (*n* = 7 vs 11, mean = 26.5 vs 18.4 months, respectively, *p* = 0.596), thus suggesting fair comparison. Of note, the latest Best Practice Advice from the American Gastroenterological Association was to defer DAA therapy 4–6 months after HCC treatment, to confirm complete and durable HCC response [38]. Details of the individual and HCC recurrence characteristics are shown in the Supplementary Table 1. None of the HCC recurrence had major vessel or extra-hepatic involvement. All of them were within the Milan criteria. The mean time from the last imaging confirming the absence of active HCC before DAA therapy till the DAA start date was 8.1 months. The mean time from the start of DAA therapy till development of HCC recurrence was 10.8 months.

As shown in Table 5, analysis of the clinical characteristics showed that patients who developed HCC recurrence had a marginally-significant higher BMI, compared to those who did not develop HCC recurrence (*p* = 0.049). The two groups did not have significant difference in terms of gender, age, presence of diabetes mellitus, genotypes, liver stiffness or SVR rate.

**Table 5:** Sub-group analysis for patients with a history of curative HCC treatment and confirmed complete radiologic response before DAA therapy

	Did not develop HCC recurrence after DAA therapy		HCC recurrence after DAA therapy		*p*-value
	(*n* = 11)		(*n* = 7)		
**Male**	8	(72.7)	7	(100)	0.245
**Age, year**	70.2	± 7.7	66.9	± 11.9	0.480
**BMI, kg/m**^**2**^	22.3	± 2.7	26.1	± 4.7	**0.049**
**HCV genotype**					0.736
**1a**	0		1	(14.3)	
**1b**	5	(45.5)	4	(57.1)	
**2**	2	(18.2)	0		
**3**	1	(9.1)	1	(14.3)	
**6**	3	(27.3)	1	(14.3)	
**HCV viral load before DAA therapy, ×10**^**6**^ **IU/ml**	1.9	± 2.2	1.0	± 1.2	0.860
**DAA therapy duration, weeks**	12 [12, 12]		12 [12, 12]		0.724
**Haemoglobin, g/dL**	12.9	± 1.9	13.6	± 1.9	0.413
**Platelets, ×10**^**9**^**/L**	118.5	± 44.6	121.7	± 66.1	0.930
**Creatinine, umol/L**	78.1	± 26.1	92.6	± 28.3	0.126
**ALT at the start of DAA therapy, IU/L**	96.0	± 88.9	74.3	± 64.6	0.724
**ALT at the end of DAA therapy, IU/L**	23.2	± 5.6	21.1	± 10.9	0.660
**Total bilirubin, umol/L**	16.5	± 7.7	26.9	± 14.7	0.122
**Albumin, g/L**	35.6	± 3.3	30.4	± 7.3	0.115
**INR**	1.13	± 0.15	1.15	± 0.08	0.425
**Prothrombin time, second**	12.7	± 1.8	13.0	± 1.1	0.285
**AFP at diagnosis of previous HCC before DAA therapy**	54.0	± 67.5	147.1	± 131.6	0.056
**AFP at the start of DAA therapy, ng/ml**	25.5	± 38.9	27.7	± 24.5	0.246
**AFP at the end of DAA therapy, ng/ml**	6.6	± 4.7	13.2	± 8.2	0.069
**HBV co-infection**	0		0		–
**HIV co-infection**	0		0		–
**Diabetes mellitus**	1	(9.1)	0		1
**Cirrhosis**	10	(90.9)	6	(85.7)	1
**Liver stiffness, kPa**	15.8 [11.9, 21.5]		23.7 [14.8, 26.2]		0.479
**Fatty liver**	3	(27.3)	0		0.245
**SVR 12**	11	(100)	7	(100)	–
**Evidence of portal hypertension**	6	(54.5)	5	(71.4)	0.637
**History of anti-viral treatment before DAA therapy**	3	(27.3)	2	(28.6)	1
**HCV infection suspected to be via previous intra-venous drug abuse**	4	(36.4)	2	(28.6)	1
**HCV infection suspected to be via previous blood transfusion**	1	(9.1)	0		1
**Previous HCC nodule(s) before DAA therapy:**					0.412
**Single**	10	(90.9)	6	(85.7)	
**Multiple**	0		1	(14.3)	
**Maximum HCC Nodule size before DAA therapy, cm**	3.0	± 1.7	2.4	± 1.5	0.364
**Previous HCC Treatment before DAA therapy:**					0.141
**Resection**	2	(18.2)	2	(28.6)	
**Ablation ± TACE**	9	(81.8)	5	(71.4)	
**Time between last HCC treatment till latest imaging before DAA, months**	12.6	± 23.3	18.4	± 27.8	0.930
**Time between last HCC treatment till DAA start date,months**	18.4	± 31.1	26.5	± 31.8	0.596
**Time between latest imaging before DAA till DAA Start date, months**	5.7	± 8.0	8.1	± 8.1	0.375

Data was expressed as count (%), mean ± SD or median [IQR]

*AFP* alfa-fetoprotein, *ALT* alanine transaminase, *BMI* Body-mass index, *DAA* direct-acting anti-viral, *HBV* hepatitis B virus, *HCC* hepatocellular carcinoma, *HCV* hepatitis C virus, *HIV* human immunodeficiency virus, *INR* international normalized ratio, *SVR* sustained virological response

Bold *p*-values are < 0.05, i.e. statistically significant

### Other liver-related complications

24 (8.6%) patients newly developed other liver-related complications after DAA therapy. The details are shown in Table 2.

**Fig. 5:**
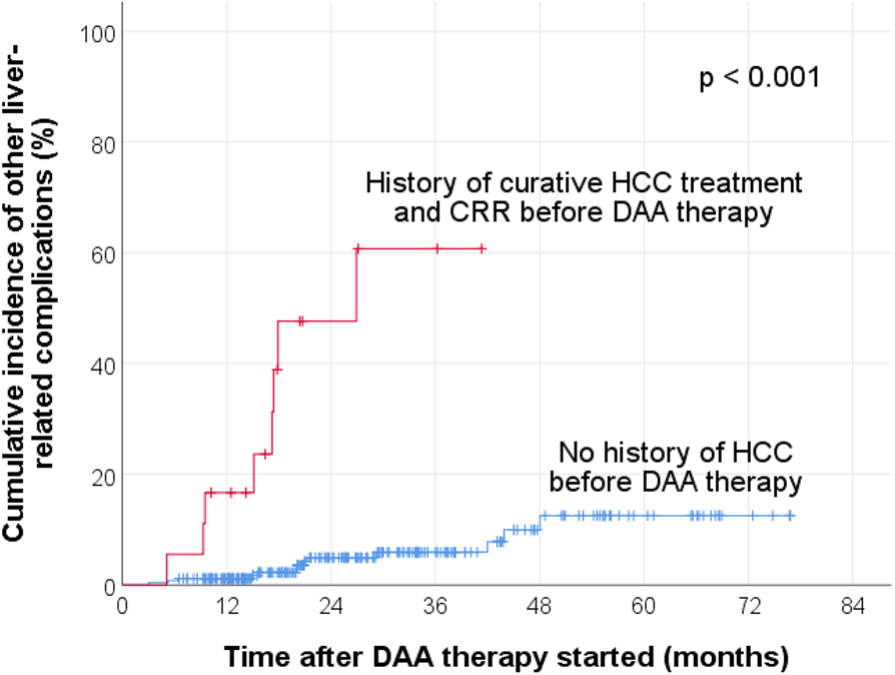
Cumulative incidence of other liver-related complications after the initiation of DAA therapy, stratified by the presence or absence of previous HCC before DAA therapy. CRR, complete radiologic response

**Fig. 6:**
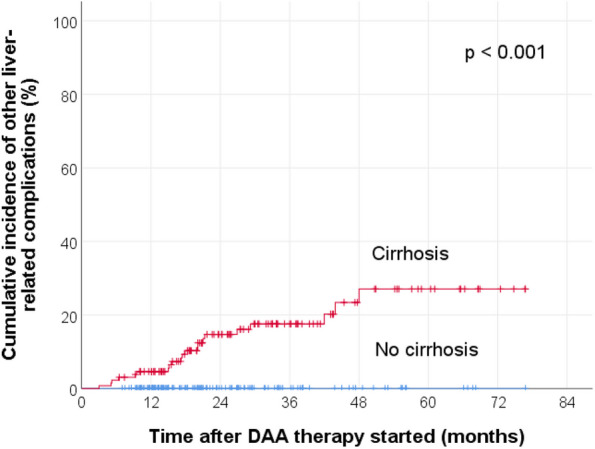
Cumulative incidence of other liver-related complications after the initiation of DAA therapy, stratified by the presence or absence of cirrhosis

These complications significantly occur more often in those who developed de-novo HCC occurrence or HCC recurrence, than those who did not develop new HCC after DAA therapy (*p* < 0.001). The 1-year cumulative incidence of other liver-related complications was 1.6% (95% CI 0–2.4%) for patients without a history of HCC before DAA therapy, and it was 19.6% (95% CI 0–33.9%) for patients with a history of curative HCC treatment with subsequent complete radiologic response before DAA therapy. The KM curves are shown in Fig. 5 (log-rank test *p* < 0.001). The 1-year cumulative incidence of other liver-related complications was 0% for patients without cirrhosis, and it was 4.6% (95% CI 1.0–8.2%) for patients with cirrhosis. The KM curves are shown in Fig. 6 (log-rank test *p* < 0.001).

## Discussion

This study has several key findings. First, the 1-year post-DAA therapy cumulative HCC recurrence rate of 30.9% is high. Despite treatments such as surgical resection and thermal ablation with curative intent, HCC is well-known to have a high recurrence rate, with an annual rate up to 15–20% [12, 13]. Such a high recurrence rate is not commonly observed in other malignancies. Similar to this study’s finding, a recent meta-analysis found that 30% (CI, 26–34%) of chronic hepatitis C patients developed HCC recurrence after DAA therapy, at mean time intervals ranging from 4 to 21 months [39]. The authors also observed a tendency towards a significant difference in terms of HCC recurrence rates from different locations around the world. The HCC recurrence rates were 23% (CI, 17–28%) from European studies, whereas those from Asian studies were 33% (CI, 27–40%). This observation might be explained by the following. Apart from the difference in inherent genetic background from different ethnicities, it was noted that there is wide variation in HCV genotype distribution from different geographical regions [40]**.** Genotype 1b, which is known to be associated with an increased risk of HCC development compared to other genotypes, is much more frequent in Asia than in western Europe [40–42]. In fact, the commonest genotypes in Hong Kong and in the current study were, in descending frequency, 1b, 6, and 3, which are all associated with a higher risk of HCC [2, 37, 43]. Furthermore, a cohort study conducted in 464 patients with chronic hepatitis C and cirrhosis in the United States found that HCC risk is increased 4-fold in Asians compared with Caucasians (adjusted odds ratio, 4.3, 95% CI, 2.1–9.0 for men; and 4.6, 95% CI, 1.2–18.5 for women) [44]. As demonstrated in Fig. 2a and Table 3**,** history of previous HCC despite achieving CRR by curative treatment before DAA therapy is a very strong risk factor for future HCC development. This highlights the importance of adherence to the recommended intervals of close HCC surveillance, especially within the initial 2 years after treatment. Various international guidelines have suggested that HCC surveillance should be done every 3 to 4 months for the first year, and then every 6 months for 3 years, by CT or MRI, along with AFP monitoring [11, 45, 46].

Second, this study was able to identify several other factors strongly associated with the development of HCC after DAA therapy (Table 3). These include, in particular, the presence of cirrhosis, evidence of portal hypertension, high AFP level at the start and end of DAA therapy. It has been shown previously that cirrhosis or markers of portal hypertension are consistently associated with HCC development [47, 48]. As mentioned above, ROC curve analysis showed that the optimal cut-off levels of AFP for predicting HCC development were ≥ 10.5 ng/mL and ≥ 5.6 ng/mL, at the start and end of DAA therapy, respectively. Various different cut-off levels of AFP were found in previous studies. In a prospective multicenter cohort study including 3012 patients with chronic HCV infection, Nakano et al. found that a higher AFP level before DAA therapy (at a cut-off value of AFP ≥ 5.4 ng/ml), indicated a higher risk of HCC recurrence after curative treatment (*p* = 0.0047) [49]. On the other hand, in a multicenter cohort study of 1675 patients who achieved SVR following treatment with DAA therapy, Ogawa et al. showed that the 1-year cumulative de-novo HCC rates were 1.4 and 13.1% in the end-of-treatment AFP < 9.0 ng/mL group and AFP ≥ 9.0 ng/mL group, respectively (log-rank test *p* < 0.001) [50]. In another multicenter cohort study including 1174 patients, Watanabe et al. demonstrated that the cumulative incidence of HCC was significantly lower in the post-DAA-treatment AFP < 6.0 ng/mL group than in the post-DAA-treatment AFP > 6.0 ng/ml group (log-rank test *p* = 0.002) [51]. Furthermore, in a comparative study of HCC occurrence and recurrence in IFN-based and IFN-free therapies, Nagata et al. found that a higher level of post-DAA therapy AFP (> 5.4 ng/mL) was strongly associated with HCC occurrence (*p* = 0.028) [52]. Therefore, AFP levels at the start and end of DAA therapy could be useful for predicting HCC development. It has also been suggested that due to the elimination of confounding effects of liver necroinflammation after successful treatment of chronic HCV, the specificity of AFP in predicting HCC would be higher post-SVR, compared to pre-SVR [53].

Third, the development of other new liver-related complication(s) after DAA therapy was strongly associated with HCC development (*p* < 0.001). This should not come as a surprise due to the fact that these patients were at a more advanced stage of liver disease. As the KM curves in Figs. 2b and 6 show, patients with cirrhosis were more likely to develop HCC (*p* = 0.036) or other liver-related complications (*p* < 0.001), compared to those who did not have cirrhosis. The mean time from the start of DAA therapy to HCC development was 15.2 months (median, 13.3 months) whereas that to the earliest liver-related complication was 20.1 months (median, 17.6 months). Therefore, this might suggest a time interval of about 4 to 5 months from the diagnosis of HCC to the development of other new liver-related complication(s) after DAA therapy. Thus, apart from managing the HCC after diagnosis, clinicians could consider to search for other possible liver-related complications, if investigations such as upper endoscopy had not been done previously.

**Fig. 7:**
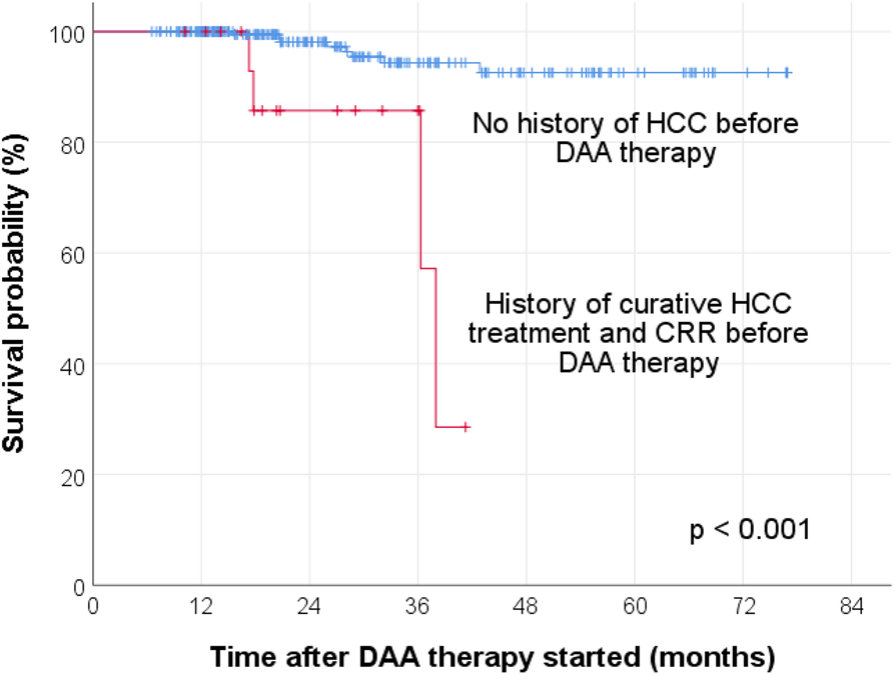
Survival probability after the initiation of DAA therapy, stratified by the presence or absence of HCC before DAA therapy. CRR, complete radiologic response

Fourth, as demonstrated in Figs. 5 and 7, history of previous HCC despite achieving CRR following curative treatment was a strongly significant factor associated with other new liver-related complications and all-cause mortality after DAA therapy, respectively. Once again this is likely due to the underlying advanced liver disease or cirrhosis in the first place. Importantly, it should be noted that liver decompensation is the main driver of mortality in HCV-infected cirrhotic patients even with successfully treated early HCC [54].

One of the strengths of this study is that most (96%) of the included patients have an imaging of the abdomen performed before the start of DAA therapy. This reduces the chance of misclassification into different sub-group in the subsequent analyses. Also, the time intervals from the last imaging before DAA therapy to the start of DAA therapy were similar in various sub-groups (*p* > 0.05), as shown in Tables 1 and 5, thus ensuring fair comparison. Other strengths of the study include that patients were recruited from two different hospitals, and the stringent exclusion criteria.

This study did not confirm the statistical significance of well-known HCC risk factors such as being male, presence of diabetes mellitus, HBV co-infection, or HIV co-infection [46, 55]. This could be well due to the small sample size and hence suffering from the random error effect. Moreover, only 17.6% of the included patients have diabetes mellitus and their mean pre-DAA therapy HbA1c was 7.1%, suggesting their diabetes were reasonably well-controlled.

The availability of highly effective oral DAA therapy has revolutionized the treatment landscape of chronic hepatitis C. These regimens allow patients once considered difficult to be treated, such as those infected with genotype 3, or cirrhosis with decompensation, to achieve a high rate of SVR [56, 57]. However, the enthusiasm for eradicating HCV has been clouded by several reports observing unexpectedly high rates of de-novo HCC occurrence and HCC recurrence soon after DAA therapy. As mentioned above, in a multi-center retrospective Spanish study by Reig et al. [24], it was found that 27.6% of patients developed HCC recurrence over a median follow-up of 5.7 months after DAA therapy. A similar concern was again noted in a subsequent Italian report [23], in which Conti et al. found that in 344 cirrhotic patients treated with DAA therapy, 28.8% developed HCC recurrence and 3.2% developed de-novo HCC after a follow-up period of 24 weeks. Furthermore, in a single center retrospective study of 66 patients with HCV-related cirrhosis [58], Ravi et al. observed that 6 patients (9.1%) developed de-novo HCC during or within 6 months of DAA therapy. The HCC incidence rates from these reports all exceeded the previously known 3–7% annual risk of HCC in HCV-induced cirrhotic patients [6]. Compared with the mere estimated 1% annual risk of HCC in patients with SVR treated by IFN-ribavirin combo therapy [59], the observed high incidence rates of HCC development from these reports, especially soon after DAA therapy, have fueled debate in the past few years over the safety concern of DAA therapy.

However, one must be careful when directly comparing studies that involve chronic hepatitis C patients treated with traditional IFN versus DAA therapy. Selection bias would be an important consideration as DAA therapy allows a much wider spectrum of patients, especially those with advanced liver disease who previously would be contra-indicated for IFN, to be treated. Needless to say, patients with advanced liver disease are at higher risk of developing HCC per se [60]. In the current study, a relatively high proportion of the included patients had cirrhosis (131 out of total 279 patients, i.e., 47%). Moreover, 65 out of the 131 (i.e., 49.6%) patients with cirrhosis had evidence of portal hypertension, which is another risk factor of HCC. Therefore, HCC development after DAA therapy could have occurred anyway but in temporal relationship to the anti-viral therapy. This highlights the importance of continuous HCC surveillance despite SVR in patients with advanced fibrosis or cirrhosis.

### Immune surveillance

There is indeed immuno-pathogenic data that would make the hypothesis of increased HCC risk following DAA therapy plausible, as follows.

DAA therapy eradicates the virus in a mere couple of weeks in most of the cases. It has been suggested that the rapid reduction in viral load causes a misbalance of HCV-stimulated immune control, which could potentially lead to proliferation of small neoplastic clones [61, 62]. HCC is an inflammatory-model cancer, arising from the interplay between the immune-mediated oxidative stress in the liver microenvironment and the direct carcinogenic effects of HCV-proteins [63, 64]. The liver microenvironment and the HCV-induced inflammation play an important role in chronic liver injury and tumour initiation [65]. The immune system has an important function in recognizing tumour cell antigens and suppressing tumour growth [66]. Tumour mass dormancy is achieved from the fine equilibrium between proliferation and death of cancer cells [67]. This equilibrium results from balancing the pro-tumour and anti-tumour functions of the immune system, known as the immune surveillance [68].

Chronic hepatitis C infection activates an intra-hepatic immune response, causing increased expression of IFN-stimulated genes and activation of natural killer cells [69], which are the first line of defense against viral infection. Natural killer cells are the most prevalent innate immune cell in the liver and provide anti-tumour function through direct lysis of cancer cells [70]. The innate and adaptive immune response to chronic HCV infection achieve anti-tumour function by i) the anti-proliferative and immuno-modulatory properties of IFNs; and by ii) the cytotoxic effects of natural killer cells and CD8 + T cells [63]. In chronic hepatitis C infection, the virus evades both the innate and adaptive immune system by releasing a number of mediators, causing impaired viral clearance and chronicity of the infection [71]. It has been suggested that with IFN-free DAA therapy, the brutal and rapid eradication of HCV causes reconstitution of the innate immunity and downregulation of type II and III IFN, their receptors, and IFN-stimulated genes [72, 73]. Therefore, the reduced IFN activation and the abrupt withdrawal of immune surveillance may in turn allow proliferation of precancerous lesions or isolated small neoplastic clones and thus HCC development after DAA therapy [74].

Having said that, there are two main models for HCC recurrence to occur after the so-called complete response following curative treatment. The first is through dissemination of tumour cells from the original HCC, known as intra-hepatic metastasis; and the second model is through new tumour(s) arising in the genetically altered liver microenvironment due to the underlying cirrhosis, known as multicentric carcinogenesis. To distinguish the two models of HCC recurrence, the amount of time between curative treatment and recurrence could be used as a reference. For the former, early recurrence, i.e. within 1–2 years, usually occurs due to intra-hepatic metastasis; whereas for the latter, recurrence usually occurs more than 2 years after curative treatment. In the current study, the mean time from the last HCC treatment till diagnosis of HCC recurrence after DAA therapy was 37.4 months, suggesting the HCC recurrences were mainly due to metachronous HCC.

### A debate near the end?

Although it is well known that the risk of HCC, especially in cirrhotic patients, remains despite SVR [11], it is essential to clarify the role of DAA therapy, if any, in the development of HCC.

More recently, several large studies have provided reassurance on the safety of DAA therapy – seemingly bringing the above-mentioned “debate” to an “end”. Ioannou et al. showed that DAA-induced SVR was associated with a 71% reduction in HCC risk, and concluded that DAA therapy was not associated with increased HCC risk compared to treatment with IFN [75]. In a multi-center prospective study including 1927 HCV-infected cirrhotic patients treated with DAA therapy from Italy, LLeo et al. demonstrated a significant reduction in HCC incidence and recurrence in patients with SVR, compared with those who did not achieve SVR [34]. In another multi-center prospective study of 2249 patients with hepatitis C associated cirrhosis, Calvaruso et al. also found that DAA-induced SVR decreased the incidence of HCC over a mean follow-up of 14 months [35]. In addition, in a massive multi-center prospective cohort study in France that included 7344 patients treated with DAA therapy, compared with 2552 patients without treatment over a mean follow-up period of 33.4 months, Carrat et al. concluded that DAA therapy was associated with a reduced HCC risk (adjusted HR = 0.66, 95% CI: 0.46–0.91) and all-cause mortality (adjusted HR = 0.48, 95% CI: 0.33–0.70) [76]. Therefore, mounting evidence from various large, prospective studies have confirmed the beneficial effect of achieving SVR via DAA therapy in reducing HCC risk. In the current study, the 1-year cumulative incidence of de-novo HCC development after the initiation of DAA therapy was 0.8% (95% CI 0–2%), which is even lower than the 1.1% found by Kanwal et al. in a retrospective cohort study of 18,076 SVR patients with a mean 2.9 years of follow-up [77].

### Limitations

This study has several limitations. First, there were only 279 patients included in the study, which was conducted retrospectively. A small sample size could cause the results more vulnerable to the random error effect. It is well known that lack of SVR is a significant predictor of HCC development [11], somehow all the patients who subsequently developed de-novo HCC occurrence or HCC recurrence achieved SVR after DAA therapy. Furthermore, 6.3% (21 out of 333) of the consecutive patients identified initially were excluded from analyses as they were either lost to follow-up or had no evaluable SVR data, hence adding uncertainty to the overall SVR rate of 98.9%. Although the sample size was small, the genotype distribution of this study was similar to that of the Hong Kong population and therefore the findings should be somewhat representative. Of all the included patients in this study, 53.8% were infected with genotype 1, 35.7% were genotype 6 and 7.9% were genotype 3. This is comparable to the findings in a territory-wide population-based study of chronic hepatitis C patients in Hong Kong by Hui et al. [2], in which the authors found that 48.8% were genotype 1, 33.6% were genotype 6 and 10.8% were genotype 3. Future prospective studies of larger sample size can be conducted in the local population to better guide HCC risk stratification according to different sub-groups. Second, this study lacks an untreated controlled arm. The ideal method to determine whether DAA therapy affects HCC risk would be to randomize chronic hepatitis C patients to treatment with DAA therapy versus no treatment, with a subsequent long follow-up period. However, given that DAA therapy has now become the standard treatment for chronic hepatitis C and with its increasing availability, such a study design would be increasingly difficult or even considered unethical. One way of conducting such studies would be to compare patients who received DAA therapy as part of their routine clinical care to those who did not receive DAA therapy for whatever reason. Third, there are confounding factors associated with the progression of liver disease and HCC development unadjusted, such as alcohol intake, tobacco exposure, or environmental toxins. Unfortunately, due to the retrospective nature of the current study and missing data from the medical records, it would be difficult to account for these confounding factors.

## Conclusion

In the current study, over a follow-up period of 23.4 months after the initiation of DAA therapy in chronic hepatitis C patients in Hong Kong, the 1-year cumulative incidence for de-novo HCC development and HCC recurrence were 0.8 and 30.9%, respectively. Clearly, the risk of early HCC recurrence, despite achieving complete radiologic response following previous curative HCC treatment and achieving SVR following DAA therapy, is high. This highlights the importance of regular and frequent HCC surveillance, especially within the initial 2 years after treatment. Cirrhosis, evidence of portal hypertension, higher bilirubin, lower platelet count, lower albumin, and more advanced age, are risk factors for HCC development after DAA therapy. Higher AFP levels, with cut-off at 10.5 ng/mL and 5.6 ng/mL, at the start and end of DAA therapy respectively, can be useful in stratifying the risk of HCC development.

### Supplementary Information

**Additional file 1.**

## Data Availability

The raw data used in this study can be accessed via the electronic medical record system (Clinical Management System) of the Hospital Authority of Hong Kong. The dataset used during the current study is available from the corresponding author on reasonable request.
