# Supplementary material for: Evaluation of patients treated with direct-acting anti-viral therapy for chronic hepatitis C and their risk of hepatocellular carcinoma in Hong Kong
Source: BMC Gastroenterol. 2024 Jan 25;24:49. doi: 10.1186/s12876-023-03099-2 (PMC10811862; doi:10.1186/s12876-023-03099-2)
Supplement: Supplementary file 1 — Additional file 1. [file 12876_2023_3099_MOESM1_ESM.docx]

**Supplementary Table 1.** Individual characteristics of patients with HCC development after DAA therapy.

| **Patient** | **Sub-group** | **Sex** | **Age** | **AFP, ng/mL** | | **Cirrhosis** | **Child Pugh score at start of DAA** | **Evidence of portal hypertension** | **No. of previous HCC nodule(s) before DAA** | **Largest previous HCC nodule before DAA, cm** | **BCLC stage of previous HCC before DAA** | **Treatment of previous HCC before DAA** | **Time from last HCC treatment till HCC diagnosed after DAA, months** | **No. of HCC nodule(s) after DAA** | **Largest HCC nodule after DAA, cm** | **BCLC stage of HCC after DAA** | **Time from DAA start till HCC diagnosed, months** |
| --- | --- | --- | --- | --- | --- | --- | --- | --- | --- | --- | --- | --- | --- | --- | --- | --- | --- |
|  |  |  |  | **At start of DAA** | **At end of DAA** |  |  |  |  |  |  |  |  |  |  |  |  |
| 1 | De-novo HCC | M | 70 | 275 | 7.9 | no | / | no | / | / | / | / | / | 1 | 1.4 | 0 | 0.2 |
| 2 | De-novo HCC | M | 58 | 71 | 4.7 | yes | 5 | no | / | / | / | / | / | 1 | 4.4 | A | 27.1 |
| 3 | De-novo HCC | F | 71 | 107 | 9.9 | yes | 6 | yes | / | / | / | / | / | 1 | 2.4 | A | 18.2 |
| 4 | De-novo HCC | M | 62 | 7.5 | 6.2 | yes | 5 | no | / | / | / | / | / | 1 | 3 | A | 20.1 |
| 5 | De-novo HCC | M | 65 | 3.7 | 4 | yes | 6 | yes | / | / | / | / | / | 1 | 2.8 | A | 8.8 |
| 6 | De-novo HCC | F | 82 | 12 | 8.3 | no | / | no | / | / | / | / | / | 1 | 2.3 | A | 13.3 |
| 7 | De-novo HCC | F | 69 | 7.9 | 8.3 | yes | 5 | yes | / | / | / | / | / | 1 | 1.5 | 0 | 18.4 |
| 8 | De-novo HCC | M | 61 | 30 | 8 | yes | 5 | yes | / | / | / | / | / | 2 | 1.4 | A | 45.5 |
| 9 | HCC recurrence | M | 67 | 15 | 16 | yes | 5 | yes | 1 | 1.6 | 0 | ablation + TACE | 12.3 | 1 | 1.8 | 0 | 9.9 |
| 10 | HCC recurrence | M | 79 | 13 | 5.6 | yes | 6 | yes | 1 | 2 | 0 | resection | 15.8 | 1 | 1.5 | 0 | 1.9 |
| 11 | HCC recurrence | M | 49 | 62 | 27 | yes | 8 | yes | 1 | 2.3 | A | resection | 11.6 | 2 | 2.4 | A | 7.3 |
| 12 | HCC recurrence | M | 56 | 11 | 4.9 | yes | 8 | yes | 1 | 1 | 0 | ablation + TACE | 97.0 | 1 | 4 | A | 19.1 |
| 13 | HCC recurrence | M | 66 | 13 | 11 | no | / | no | 2 | 5.6 | B | ablation + TACE | 93.3 | 1 | 3 | A | 26.8 |
| 14 | HCC recurrence | M | 68 | 65 | 20 | yes | 6 | no | 1 | 1.7 | 0 | ablation + TACE | 13.6 | 1 | 1.4 | 0 | 10.0 |
| 15 | HCC recurrence | M | 83 | 15 | 7.6 | yes | 9 | yes | 1 | 2.8 | A | ablation + TACE | 18.0 | 1 | 2.3 | A | 0.7 |

AFP, Alpha-fetoprotein. BCLC, Barcelona Clinic Liver Cancer. CRR, Complete Radiologic Response. DAA, Direct-acting-anti-viral. SVR, Sustained Virological Response.
